# Inferring symmetric and asymmetric interactions between animals and groups from positional data

**DOI:** 10.1371/journal.pone.0208202

**Published:** 2018-12-12

**Authors:** Edward Hollingdale, Francisco Javier Pérez-Barbería, David McPetrie Walker

**Affiliations:** 1 Department of Mathematics and Statistics, University of Western Australia, Nedlands, Perth, WA, Australia; 2 Game and Livestock Resources Unit, University of Castilla-La Mancha IDR IREC, Albacete, Spain; Arizona State University & Santa Fe Institute, UNITED STATES

## Abstract

Interactions between domestic and wild species has become a global problem of growing interest. Global Position Systems (GPS) allow collection of vast records of time series of animal spatial movement, but there is need for developing analytical methods to efficiently use this information to unravel species interactions. This study assesses different methods to infer interactions and their symmetry between individual animals, social groups or species. We used two data sets, (i) a simulated one of the movement of two grazing species under different interaction scenarios by-species and by-individual, and (ii) a real time series of GPS data on the movements of sheep and deer grazing a large moorland plot. Different time series transformations were applied to capture the behaviour of the data (convex hull area, *k*^*th*^ nearest neighbour distance, distance to centre of mass, Voronoi tessellation area, distance to past position) to assess their efficiency in inferring the interactions using different techniques (cross correlation, Granger causality, network properties). The results indicate that the methods are more efficient assessing by-group interaction than by-individual interaction, and different transformations produce different outputs of the nature of the interaction. Both species maintained a consistent by-species grouping structure. The results do not provide clear evidence of inter-species interaction based on the traditional framework of niche partitioning in the guild of large herbivores. In view of the transformation-dependent results, it seems that in our experimental framework both species co-exist showing complex interactions. We provide guidelines for the use of the different transformations with respect to study aims and data quality. The study attempts to provide behavioural ecologists with tools to infer animal interactions and their symmetry based on positional data recorded by visual observation, conventional telemetry or GPS technology.

## Introduction

Assessing animal interactions in species assemblages is key for the understanding of competition and facilitation processes [1, 2], species conservation [3], habitat use [4–6], group-living decision-making mechanisms [7, 8], energy expenditure [9] and spread of zoonotic diseases [10, 11]. The booming economies of developing countries have increased pressure for space in natural areas, while in developed countries rural abandonment is causing an increase in numbers of some wild species. As a result, interactions between domestic and wild species has become a global problem of growing interest [3, 12]. This is especially important in ungulate species, as they are paramount in human development, the economy, conservation of ecosystems and climate change [13, 14].

The affordability and precision of Global Position Systems (GPS) have made possible to record the spatial movement of a variety of terrestrial and marine taxa [15]. Despite the large development on GPS technology and integrated behavioural and environmental recording sensors [15], there is need for developing analytical protocols to efficiently use this type of information to unravel intra- and inter-species interactions [16, 17].

Extracting animal interaction from time series of animal movement is inherently complicated because of (i) the large number of potential interactions when many animals are under study, (ii) the vast number of spatial records when using GPS data of marked animals, (iii) the arbitrary choice of defining an interaction based on positional data, (iv) the problem of establishing the symmetry of the interaction, (v) the assessment of whether the interaction takes place at individual, social or taxonomic level, and (vi) the almost inevitable presence of missing records within positional data time series. A number of methods have been used in the animal behaviour literature to deal with some of these issues, but there are not well-established protocols for accounting for all. Methods which can be used to establish animal interactions include those based on the area (range) an animal uses over a fixed period of time [18, 19]; a second approach is based on spatial proximity, such as metric or topological distance, to other animals [17, 20]. Based on the size or overlap of the animal range in comparison with those of others, or the proximity to other animals, interaction between two animals, social units or species can be established across time by fixing arbitrary thresholds of interaction [17]. The construction of scalar time series to infer interactions can be undertaken by either using data [19, 21] or functions, such as black box radial basis, which can then be fitted to data [2, 9, 22–25].

In order to infer from spatial movement processes of competition and/or facilitation it is crucial to assess the direction or symmetry of the interaction, that is, does A drive B, or B drive A? (an asymmetric interaction); or rather movement of A and B are symmetrically coupled. There have been attempts of inferring the symmetry of the interactions between grazing species (sheep and deer) but with inconclusive results [2, 9, 19]. The direction of the interaction can be assessed by testing for causality. Many approaches exist to examine possibly causal relationships between two time series. Among these are cross-correlation and Granger causality [26], which have been used to analyze dependence between factors in a variety of fields, such as finance, econometrics [27–29], neuroscience [30] and parasitology [11].

Cross-correlation is a measure of similarity of two time series as a function of the displacement of one over the other. It is not a measure of causality but strong correlation is suggestive of a possible causal relationship between time series to be investigated further such as with Granger causality. Granger causality is determined by testing whether one variable can be better predicted when including information from another [26, 31]. Inherent to this approach is the assumption that a variable can be predicted using information of past values of the same variable. Defining interaction causality across pairs of animals does not solve the problem of unravelling the complexity of multiple interactions. Network theory is well-suited for summarizing complex relationships between subjects [32]. Indeed, if the causality structure is clear then one can consruct causality trees as is done in [33] for sequences of mobile phone calls. Complex interactions can be represented as a network in which vertices are subjects (animals, social units, species, etc.) connected by edges that represent interaction and direction. Network theory has been successfully used to model animal aggregation and swarming behavior based on time series of positional data. For example, in studies to understand the drivers and dynamics of murmurations and flocking of pigeons and starlings [20, 34, 35], schools of fish [23, 36], collective decisions in intermittent flocking, sexual segregation in sheep [19, 21], and sheep and deer interaction during grazing [4, 9].

Furthermore, scalar time series to infer species interactions have also been constructed using binary behavioural data, such as grazing activity budgets (resting vs. grazing) to unravel intra-species sexual synchronicity as a potential explanation for spatial and social segregation [16, 19, 37].

The aims of this study is to assess the efficiency of different methods to infer interactions and their symmetry between individual animals, social groups or species. To further aid interpretation of how meaningful any uncovered evidence of interaction is, we first test the methods on simulated positional data extracted from artificial models of animal movement patterns, then we apply the methods to real GPS datasets of the spatial positions of grazing sheep and deer in a moorland area. The study attempts to provide behavioural ecologists with tools to infer animal interactions and their symmetry based on positional data recorded by visual observation, conventional telemetry or GPS technology.

## Materials and methods

### Animal data

We used information of the spatial positions of 8–23 Scottish blackface ewes (*Ovis aries*) and 7–18 red deer hinds (*Cervus elaphus scoticus*) (Table 1) fitted with GPS collars of the study [9]. The experiments were carried out between August (2006) and December (2008) in North East Scotland in a stock-proof fenced plot of 1.04 km^2^ located in a moorland area, dominated by a mosaic of heather-grass vegetation. These animals were part of a group of 20 red deer adult hinds and 60 ewes that freely grazed the plot under no human disturbance.

**Table 1:** Time series and dates of GPS fixes, number of sheep and red deer used in this study.

	2006-S1	2006-S2	2006-S3	2008-S1	2008-S2	2008-S3	2008-S4	2008-S5
Length (h)	950	688	1552	1554	2732	1508	668	1458
No. sheep	13	13	8	23	23	23	23	23
No. deer	18	18	7	17	17	17	17	17
Start date	19/08/2006	05/10/2006	06/11/2006	01/01/2008	19/03/2008	17/07/2008	23/09/2008	01/11/2008
End date	28/09/2006	03/11/2006	10/01/2007	05/03/2008	10/07/2008	17/09/2008	20/10/2008	31/12/2008

GPS collars weighed 330 g, which was between 0.4% and 0.7% of the winter average body mass of the hinds and sheep, respectively (0.5% and 0.9% of the lightest hind and sheep), a negligible extra load to the animals. Animals were gathered every 6 months to download the information from the GPS collars, change the collars’ batteries, and at this time both deer and sheep received the standard preventative veterinary treatments used on the farm. On a regular basis, observations from a distance were carried out to assess the welfare of the animals and no apparent discomfort due to GPS collars fitting was observed in the animals, which was confirmed by close inspection of the neck around the collars every time that GPS units’ batteries were changed. After the experiment the collars were removed and the animals inspected by a veterinary surgeon before being returned to the main herd/flock. The study did not involve endangered or protected species, and GPS collar deployment on sheep or farmed red deer is not a regulated procedure under the revised Animals (Scientific Procedures) Act 1986 (See, https://protect-eu.mimecast.com/s/GYVbCk59DIAymBI2QAnR?domain=gov.uk.) that came into force on 1 January 2013 and that complies with European Directive 2010/63/EU on the protection of animals used for scientific purposes. Furthermore, the experiment was reviewed by the Animal Welfare and Ethical Review Body (AWERB) of the scientific establishment.

Time acquisition interval of positional fixes was 1 h, on the hour, and 95% of the fixes were within a 14 m radius of their actual position [9]. We selected eight time segments of length between 668 h and 2732 h (Table 1), in which there were almost continuous hourly fixes for sheep and deer, and occurrences of missing values (<2%) were linearly interpolated. We tested the robustness of this interpolation approach by selecting a contiguous sequence of observations with no missing data and randomly removed an observation. We then interpolated the value of this missing observation and compared how the time series transformations (see below) were affected. We found that linear interpolation compared to higher-order interpolation methods produced, on average, the smallest differences between the full data transformation and the interpolated transformation.

### Analytical design

**Fig 1:**
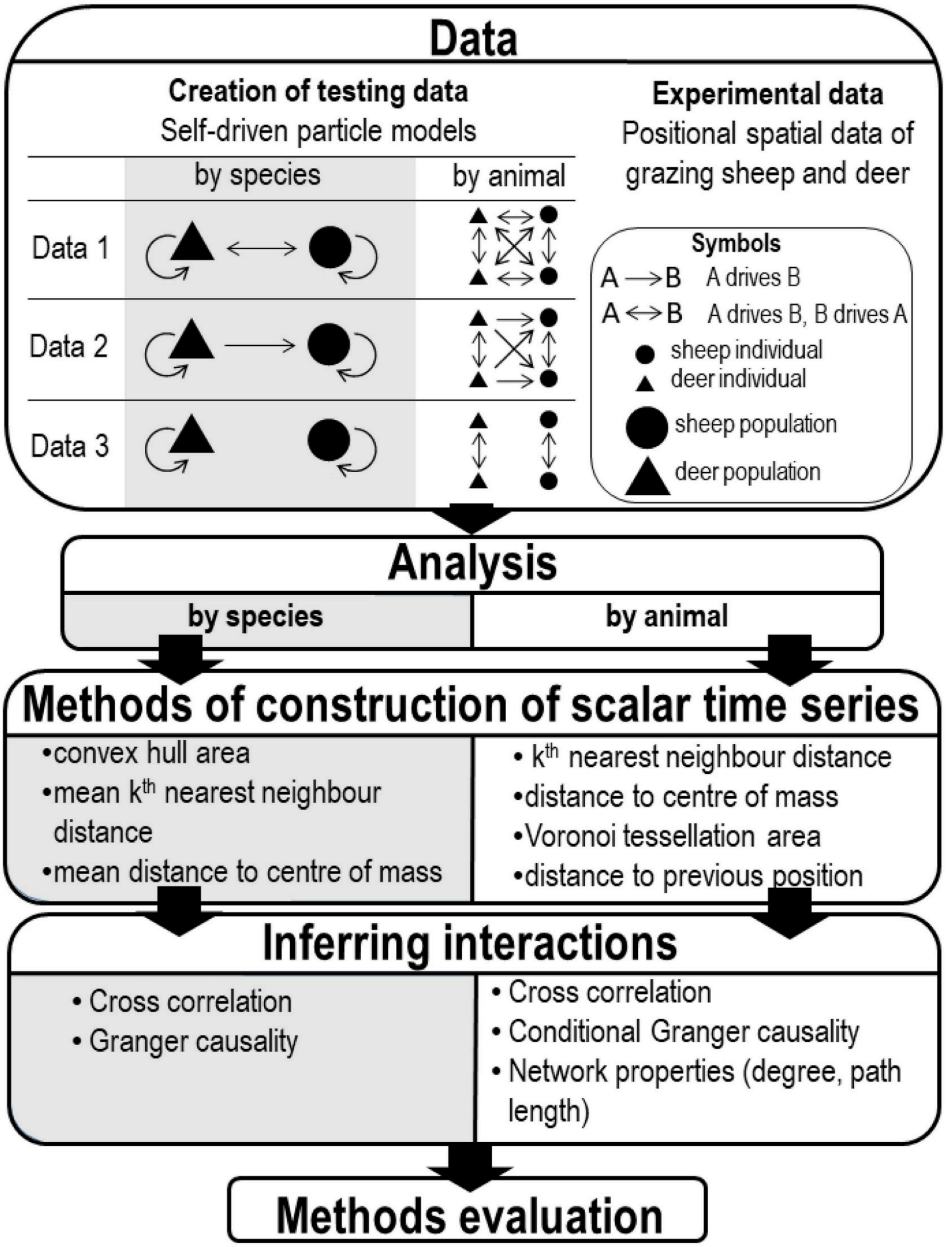
Analytical design. Analytical design to extract species interaction from time series of positional data.

We proceeded by (i) creating testing data of simulated spatial positions of sheep and deer using modified versions of [22] and [23] models on self-driven particles; (ii) transforming the testing animal position data into scalar time series of intra- and inter-species interactions using different methods from the literature (convex hull area CHA, mean *k*^*th*^ nearest neighbour distance MDNN, mean distance to centre of mass MDCM, distance to centre of mass DCM, Voronoi tessellation area VTA); (iii) testing inter-species (inter-groups) interactions by computing the cross-correlation between the transformed scalar time series and also testing for Granger causality (1969); (iv) at an individual level significant interactions between subjects were conveniently represented by a complex network whose network properties (degree, geodesic path length) helped to summarize and infer animal interactions; results obtained in (iii) followed by (iv) aided selection of the methods in (ii) that best reflected the simulated interactions; and finally (v) applying points (ii), (iii) and (iv) to our experimental data on sheep and deer movements (Fig 1, Table 2). By transforming the positional data to scalar time series the complexity of the data was reduced, making analysis possible, while the information to understand and extract meaningful interaction was maintained.

**Table 2:** Methods of constructing scalar time series from positional data of species using three data sets (Data 1, 2, 3, Fig 1) constructed using a modified Couzin model, each with different embedded intra- and inter-species interactions to test the following hypotheses *H*_*i*_. D: deer; S: sheep; *H*_1_. *D* → *S*: deer drives sheep; *H*_2_. *S* → *D*: sheep drives deer; *H*_3_. *D* ↛ *S*, *S* ↛ *D*: no interaction between species; ✔: results support hypothesis; ✖: results do not support hypothesis. Timespan: number of hours within spatial data area pooled for analysis (GPS fix acquisition time = 1 h). *k*: number of nearest neighbours in the analysis.

method	timespan	*k*	Data 1		Data 2		Data 3	
convex hull	1	all	✖	✖	✔	✔	✔	✔
	3	all	✖	✖	✔	✔	✔	✔
	5	all	✖	✖	✔	✔	✔	✔
mean distance to *k*^*th*^ nearest neighbour	1	1	✔	✔	✖	✔	✔	✔
	1	2	✖	✖	✖	✔	✔	✔
	1	3	✖	✖	✖	✔	✔	✔
	1	4	✖	✔	✖	✔	✔	✔
	1	5	✔	✖	✖	✔	✔	✔
	1	6	✔	✔	✔	✔	✔	✔
mean distance to centre of mass	1	all	✔	✔	✔	✔	✔	✔

### Simulation

**Fig 2:**
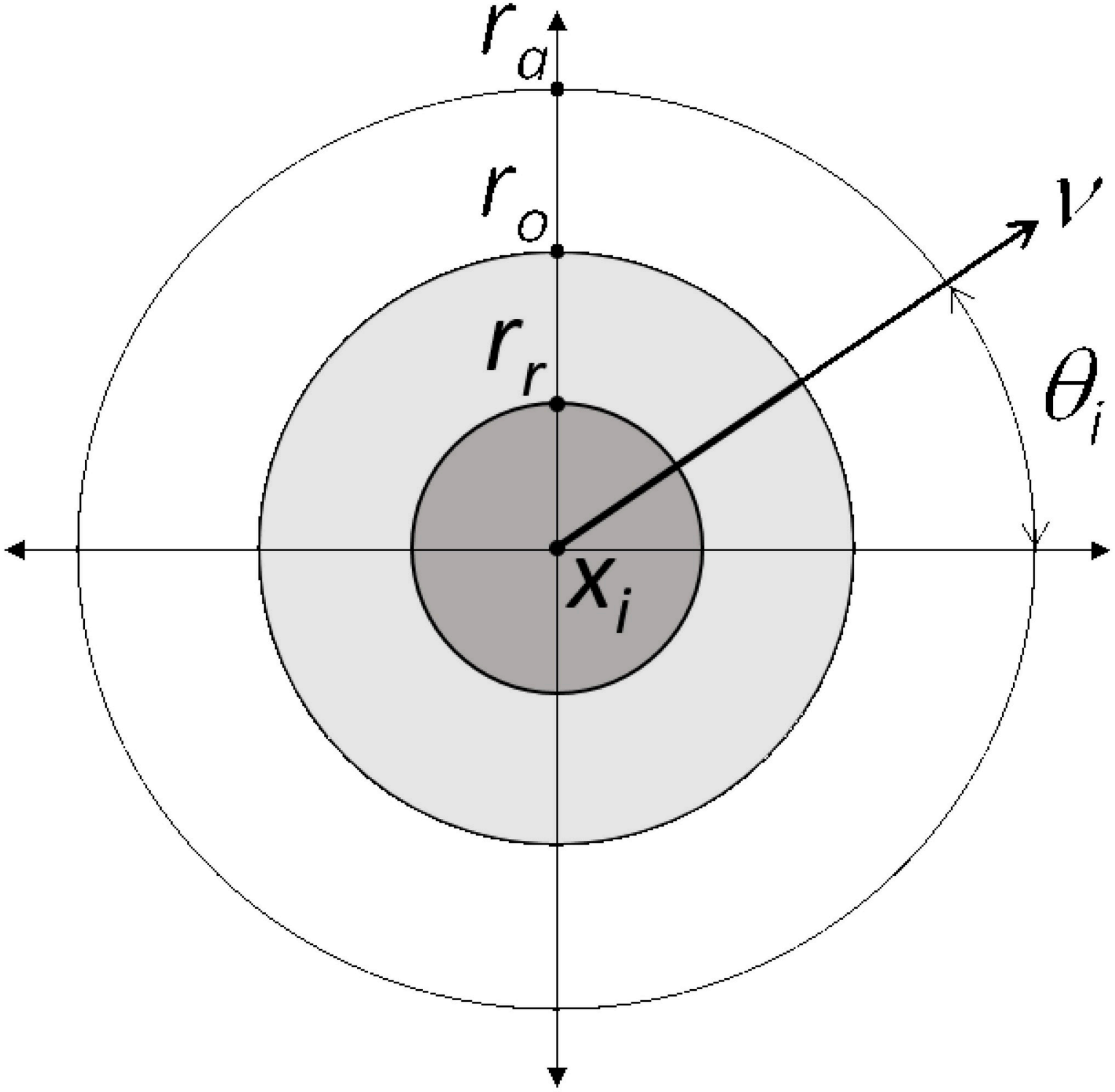
Simulation model zones. Zones of repulsion, orientation and attraction for an animal within a two dimensions Couzin model. The individual is at position *x*_*i*_ travelling in direction *θ*_*i*_ at speed *v*. The zone of repulsion is the region (in dark grey) of points within *r*_*r*_, the zone of orientation is the region (in light grey) of points between *r*_*r*_ and *r*_*o*_ of *x*_*i*_ and the zone of attraction is points between *r*_*o*_ and *r*_*a*_ of *x*_*i*_.

The model created for the purposes of generating testing data is an adaptation of the [23] model, which is itself a variation of the [22] model (hereafter Couzin model and Vicsek model). The core principle of the Vicsek model is that particles (representing animals) move with a constant velocity and align the direction of their motion with the direction of the particles around them. The Couzin model extends this by creating three different zones of: repulsion; orientation; and attraction, illustrated in Fig 2. We simplified simulation to a two-dimensional version outlined as follows: an individual animal *i* moves with velocity of a constant magnitude *v*, and its position at time *t*+ 1 is given by:

$$\begin{array}{ccc} x_{i}\left(t+1\right) & = & x_{i}\left(t\right)+v(\begin{array}{c} \text{cos}\;\theta_{i}\left(t\right)\\ \text{sin}\;\theta_{i}\left(t\right) \end{array}), \end{array}$$ (1)

where *θ*_*i*_ is the direction of movement. Individual animals attempt to maintain a minimum distance *r*_*r*_ from others (repulsion zone). If the number of animals within *r*_*r*_ of animal *i*, *n*_*r*_ is greater than 0, then *i* will move in a direction opposite to the mean direction of those individuals relative to *i*. If *n*_*r*_ = 0 (no neighbours are within the repulsion zone) then the individual observes the positions and directions of movement of the animals around it and moves according to these. If animals are within the orientation zone (distance > *r*_*r*_ and ≤ *r*_*o*_) then the individual will match its direction to the mean direction of those neighbours. If an individual animal *j* has direction *θ*_*j*_ then the direction of animal *i* will be:

$$\begin{array}{ccc} \theta_{o}\left(t+1\right) & = & \tan^{-1}(\frac{\sum_{j}\sin\theta_{j}}{\sum_{j}\cos\theta_{j}})\;\text{for}\;\text{all}\;j\;\text{where}\;r_{r}<∥x_{i}-x_{j}∥\leq r_{o}. \end{array}$$ (2)

If animals are within the attraction zone (distance ≤ *r*_*a*_ and > *r*_*o*_) then the direction of animal *i* will be the mean direction of those animals relative to animal *i*. If each animal *j* in the zone of attraction has a direction *β*_*j*_ from animal *i* then:

$$\begin{array}{ccc} \theta_{a}\left(t+1\right) & = & \tan^{-1}(\frac{\sum_{j}\sin\beta_{j}}{\sum_{j}\cos\beta_{j}})\;\text{for}\;\text{all}\;j\;\text{where}\;r_{o}<∥x_{i}-x_{i}∥\leq r_{a}. \end{array}$$ (3)

If *n*_*r*_ = 0 and both *n*_*o*_ and *n*_*a*_ (the number of neighbours in the zone of orientation and the zone of attraction, respectively) are greater than zero then the animal *i* moves in the mean direction of *θ*_*o*_ and *θ*_*a*_.

If an individual animal has no neighbours in any zone (distance to neighbours ≥ *r*_*a*_) then they continue in the same direction, that is *θ*_*i*_(*t* + 1) = *θ*_*i*_(*t*). Bounds on the positions were instituted, preventing animals from continuing to walk away from the group, if an animal reached the boundary of the simulated plot then its direction of travel was rotated by 180°. The final direction (either the opposite direction of any animals within the zone of repulsion, *θ*_*o*_, *θ*_*a*_ or the mean of the two) is then adjusted by an angle taken at random from a wrapped Gaussian distribution, with standard deviation *σ*. The probability density function of the wrapped Gaussian with a mean angle *μ* and variance *σ*^2^ is given by:

$$\begin{array}{ccc} f(\theta ;\mu ,\sigma ) & = & \frac{1}{\sigma \sqrt{2\pi }}\sum_{k=-\infty }^{\infty }\exp[\frac{-{\left(\theta -\mu +2\pi k\right)}^{2}}{2\sigma^{2}}]. \end{array}$$ (4)

Contrary to the Couzin model we assumed that the anatomy and behavior of the animals create no blind-volume in their visual field, and so no penalties are applied to any direction of movement around them.

For consistency with the experimental data, the plot size created in the model was 1 km by 1 km. To approximately match the densities observed in the data 20 sheep and 20 deer were modelled. Animals were initially randomly distributed over the plot, and the first 240 hours of movement was not included in the analysis. Time series of length 2000 hours were created. The speed, *v*, was selected as 30 m *h*^−1^ (median distance travelled by a sheep in our actual dataset). The radius of repulsion was chosen as 10 m and the radius of orientation as 40 m. In [9] it was found that the distance where sheep-deer interaction went from slight repulsion to attraction was 55 m, and for deer-sheep interaction it was 25 m. The radius of attraction was selected as 150 m, about as far as a sheep and deer can feasibly see given the hilly terrain in the actual data. Finally, the variance of the noise term, *σ* was chosen as 0.1 rad, the middle of the range of variances tested by [23].

In order to test the methods for different levels of interaction (intra-species, inter-species), additional neighbour selection requirements were set. The model took a relationship matrix *R* (a binary matrix) of interactions, only if an animal *i* and potential neighbour *j* were related (*R*_*ij*_ = 1) could neighbour *j* be used when calculating the direction of travel for animal *i*. We designated the first *m* = 20 of the *N* = 40 animals deer, and the remaining *N* − *m* sheep, so that the movements of animals of different species given a particular relationship matrix could be set. The ability to impose specific interaction scenarios via the matrix *R* was the primary motivation for using a modified Couzin model. If a specific leader-follower scenario is of interest then an alternative model of behaviour may be more appropriate, for instance, the framework described in [38] whose behaviour could be aptly analyzed using causality trees [33].

Using the described model, test data with three different relationship matrices were generated (Fig 1). Data 1 allowed all animals to interact (deer → deer, sheep → sheep, deer ↔ sheep, where the direction of the arrow indicates “influences”). This was undertaken by including all animals when applying the model to calculate the direction for a particular animal; Data 2 allowed only deer → deer, sheep → sheep, deer → sheep interactions (i.e., only the positions of deer were used to update the positions of deer, but both deer and sheep positions were used to update sheep positions); and Data 3 allowed interactions only to their own species, this was done by excluding from the calculations animals of the opposite species (i.e, deer → deer, sheep → sheep, and no inter-species influence). The output of the model runs were multivariate position data of all animals every hour for 2000 hours.

We also used these multivariate position data to construct individual animal time series and attempted to recover the exact form of the relationship matrix for each of the three test data sets above. For each time series method, a conditional Granger causality test (described below) was carried out, testing whether the time series of a particular animal Granger-caused the time series of another, conditional on the time series of the other individual animals present. In the Granger causality modelling we investigated models orders of up to *q* = 10. The p-values of these tests allowed summary complex networks of the interaction to be created—with a network edge between two individual animals (vertices of the network) being constructed if there was a significant Granger causality test p-value (*α* = 0.05). To circumvent possible type I errors (misidentification of interactions), and to prevent the creation of multitudinous networks for each set of generated data, summarized networks were used. That is a network edge representing a significant interaction between two individual animals is only created if the interaction is significant over several orders *q* of the Granger causality modelling process. Here, networks for each method and relationship structure were created with edges formed where the conditional Granger causality test p-value was significant for at least 3 values of *q*.

### Construction of scalar time series by species

Using the testing data sets of animal movement described above, we created scalar (univariate) time series from the animal positions for each species (Table 2). In total, 10 functions per species were used on the testing data. These come under three different categories: convex hull area (using hourly positions, and accumulated positions after 3 and 5 hours), mean *k*^*th*^ nearest neighbour distance (*k* = 1 to 6), mean distance to the centre of mass of the group, where groups are all sheep, or all deer, or all animals (sheep + deer).

#### Convex hull area CHA

The convex hull of a set of points is the smallest convex set that contains all of the points. The convex hull area offers a simplistic measure for the extent to which the members of a particular species aggregate or spread out in space at a particular time. If the use of space by a group of animals is affected by the proximity of another group, the changing level of clustering may be captured in the measure. Convex hull areas have previously been applied to analyze sheep positional data [21] and are used extensively in ecology as a means of estimating the home range and the utilization distributions of an individual animal or a group of animals [39–43]. CHA was calculated using the positions of all the animals of a particular species over periods of 1, 3 or 5 hours. When using 3 or 5 hour timespans the positions of each individual animal occur multiple times. The inclusion of multiple hours of positional data acts as a smoother on the constructed convex hull time series, partially offsetting any noise from individual movements, especially of outlying animals, or from short-time scale cluster-dispersion behaviours. The use of convex hulls over a longer timespan is common practice when calculating animal home-range [44] or by adding an additional time dimension and using a time-weighting [41].

#### Mean distance to *k*^*th*^ nearest neighbour MDNN

MDNN is based on two hypothesis, (i) that the closer the distance between two animals the higher the likelihood of interaction between them, and (ii) the distance itself can be an indication that an interaction has occurred, as in general, interaction would be expected to be at least at visual range to interact (i.e. excluding interactions by vocal calling and olfactory communication [45]). MDNN is calculated by finding the *k*^*th*^ nearest neighbour of the same species to an individual animal at a given time, and calculating the distance to it. The mean of these distances over a particular species, or group, is then calculated.

We define *K*(*s*, *k*, *t*) as the mean *k*^*th*^ nearest neighbour distance for sheep at time *t* for some given number of neighbours:

$$\begin{array}{ccc} K(s,k,t) & = & \frac{\sum_{i=1}^{m}∥s_{i}\left(t\right)-s_{k(k)}\left(t\right)∥}{m}. \end{array}$$ (5)

Similarly, for deer:

$$\begin{array}{ccc} K(d,k,t) & = & \frac{\sum_{i=1}^{n}∥d_{i}\left(t\right)-d_{h(k)}\left(t\right)∥}{n}. \end{array}$$ (6)

Similar measures have been previously used, for example, [2] and [9] to assess sheep and deer interactions during grazing by measuring the difference in position to each of the *M* nearest neighbours (*M* = 5 and 6, respectively). In another study [20] used the average of the positional difference vectors of the *M* nearest neighbours when modelling flocking behavior in pigeons (with *M* = 4 for a low density of pigeons and *M* = 30 for a high density). Consequently, when computing the time series *K*(*s*, *k*, *t*) and *K*(*d*, *k*, *t*) values of *k* from 1 to 6 were used.

#### Mean distance to centre of mass MDCM

Defining the centre of mass of a species as the mean position of all the members of that species gives, for sheep:

$$\begin{array}{ccc} c(s,t) & = & \frac{\sum_{i=1}^{m}s_{i}\left(t\right)}{m}. \end{array}$$ (7)

MDCM is the average distance of a member of a species to the centre of mass of that species. For sheep, this is:

$$\begin{array}{ccc} C(s,t) & = & \frac{\sum_{i=1}^{m}∥S_{i}\left(t\right)-c\left(s,t\right)∥}{m}, \end{array}$$ (8)

and, defining *c*(*d*, *t*) as for sheep but with *d*_*j*_’s instead of *s*_*i*_’s and replacing *m* with *n*, the mean centre of mass distance, for deer, is:

$$\begin{array}{ccc} C(d,t) & = & \frac{\sum_{j=1}^{n}∥d_{j}\left(t\right)-c\left(d,t\right)∥}{n}. \end{array}$$ (9)

As with the convex hull area, MDCM offers a measure of how spread out the species is. It overcomes the issue with the convex hull area of far-flung individual animals having a disproportionate effect on the value. However it does not factor in any variations in density in local neighbourhoods, as the mean *k*^*th*^ nearest neighbour distance does.

### Construction of scalar time series by individual animal

To help infer interactions at the individual animal level we, similarly, used the testing data and created scalar time series from the multivariate animal positions (Table 3). In contrast to the species level time series transformation, different methods of construction were necessary: kth nearest neighbour distance, distance to centre of mass, area of the Voronoi tessellation (as an analog for convex hull area), and distance from past position.

**Table 3:** Out-degree of summarized networks inferred using different methods of constructing scalar time series from positional data of individual animals, using three data sets (Data 1, 2, 3) constructed using a modified Couzin model, each with different embedded inter-species interactions (intra-species interaction is present and assumed and so not tested for) to test the following three interaction hypotheses *H*_*i*_. D: deer; S: sheep; *H*_1_. D↔S: deer drives sheep and sheep drives deer; *H*_2_. D→S: deer drives sheep; *H*_3_. D ↮ S: no interaction between species. Interaction is assessed by the connectedness of the inferred interaction networks, in particular, the out-degree of intra-species network edges to inter-species networks edges. Bold font used if hypothesis supported: successful detection of the interaction. Timespan: number of hours within spatial data area pooled for analysis (GPS fix acquisition time = 1 h). *k*: number of nearest neighbours in the analysis, either all other animals, or all animals of same species.

method	timespan	*k*	Data 1 (*H*_1_: *D* ↔ *S*)				Data 2 (*H*_2_: *D* → *S*)				Data 3 (*H*_3_: *D* ↮ *S*)			
			*D* → *D*	*S* → *S*	*D* → *S*	*S* → *D*	*D* → *D*	*S* → *S*	*D* → *S*	*S* → *D*	*D* → *D*	*S* → *S*	*D* → *S*	*S* → *D*
distance to centre of mass	1	all	**4.00**	**3.60**	**3.20**	**3.90**	3.55	3.1	3.00	3.35	3.85	3.95	3.00	2.65
	1	species	**3.90**	**3.55**	**3.20**	**3.50**	3.75	3.15	2.80	2.80	3.95	3.50	2.80	2.90
Voronoi tessellation area	1	all	**3.75**	**4.10**	**3.65**	**2.85**	3.35	3.35	2.30	2.90	3.55	3.55	2.10	1.90
	1	species	**8.15**	**8.95**	**1.35**	**1.20**	**8.05**	**9.35**	**1.75**	**0.45**	**7.95**	**8.35**	**1.15**	**1.30**

#### Distance to *k*^*th*^ Nearest Neighbour DNN

Following MDNN, the distance of a given animal to each of its *k* = 1 to *k* = 6 closest neighbours was computed. This offers a measure of how isolated an animal is at a particular moment in time. For each animal three different time series were created for each value of *k*: DNN of the same species; DNN of the opposite species; and DNN of the combined species.

For sheep *i*, at time *t*, these would be defined as:

$$\begin{array}{ccc} K_{s}\left(i,k,t\right) & = & ∥s_{i}\left(t\right)-s_{k(k)}\left(t\right)∥\\ K_{o}\left(i,k,t\right) & = & ∥s_{i}\left(t\right)-d_{h(k)}\left(t\right)∥,\\ K_{c}\left(i,k,t\right) & = & ∥s_{i}\left(t\right)-c_{g(k)}\left(t\right)∥ \end{array}$$ (10)

where *c*_*g*(*k*)_ is the position of the *k*^*th*^ nearest neighbour of the combined sheep and deer positions. *K*_*s*_ is the distance to members of the same species (sheep in the above example), *K*_*o*_ the distance to members of the opposite species (deer in the above example) and *K*_*c*_ the distance to neighbours of the combined sheep and deer positions. This could be adjusted for deer *j* and time *t* by replacing all instances of *s*_*i*_(*t*) with *d*_*j*_(*t*) and switching *s*_*k*(*k*)_(*t*) and *d*_*h*(*k*)_(*t*).

#### Distance to centre of mass DCM

Following computation of the mean centre of mass distance at species level, the distance of an individual animal to the centre of mass (the mean position) of its own species, the opposite species, or to the mean position of all animals, was used. This offers another measure of how isolated an individual animal is in relation to members of its own species, members of the opposite species, or to all animals. Specifically, the combined mean position at time *t* as:

$$\begin{array}{ccc} c(c,t) & = & \frac{\sum_{i=1}^{m}s_{i}\left(t\right)+\sum_{j=1}^{n}d_{j}\left(t\right)}{m+n}. \end{array}$$ (11)

The three DCM for sheep *i* at time *t* are:

$$\begin{array}{ccc} C_{s}\left(i,t\right) & = & ∥s_{i}\left(t\right)-c\left(s,t\right)∥\\ C_{o}\left(i,t\right) & = & ∥s_{i}\left(t\right)-c\left(d,t\right)∥,\\ C_{c}\left(i,t\right) & = & ∥s_{i}\left(t\right)-c\left(c,t\right)∥ \end{array}$$ (12)

where *C*_*s*_ is the DCM for the same species, *C*_*o*_ is the DCM for the opposite species, and *C*_*c*_ is the DCM to the combined species. These could be similarly defined for deer *j* at time *t* by replacing all instances of *s*_*i*_(*t*) with *d*_*j*_(*t*) and switching *c*(*s*, *t*) and *c*(*d*, *t*).

#### Voronoi tessellation area VTA

A Voronoi tessellation is a partitioning of a plane into areas of influence about a set of sites, where for each site there is a corresponding polygon of points that are closer to that site than any other [39, 43]. The area of the Voronoi polygon formed about an individual animal can be used as a measure for its isolation [39]. Voronoi tessellations for neighbourhood construction have been used successfully for characterizing flocking and school behavior in birds, fish and for animal movement prediction and modelling [36, 39, 43, 46, 47].

Two scalar time series can be produced for each individual animal based on Voronoi tessellations. The first (*V*_*s*_) is the area of the Voronoi polygon of an individual animal with the tessellation constructed using the positions of all members of the same species. This is a measure of how isolated the animal is in relation to all the members of its own species. The second (*V*_*c*_) is the area of the Voronoi polygon of an individual animal with the positions of all animals in the data set at the given time *t* used in the formation of the Voronoi tessellation. This is a measure of how isolated the animal is in relation to all the animals, including those of the opposite species.

#### Distance from past position DPP

The final method to transform animal positional data into scalar time series is the distance from a previous position of that animal. The change in position has been previously used as a model input for animal movement [20]. For a sheep *i* at time *t*, using the change in position over *T* hours, the function is:

$$\begin{array}{ccc} PD(i,T,t) & = & ∥s_{i}\left(t\right)-s_{i}\left(t-T\right)∥. \end{array}$$ (13)

It is similarly defined for a deer *j* as:

$$\begin{array}{ccc} PD(j,T,t) & = & ∥d_{j}\left(t\right)-d_{j}\left(t-T\right)∥. \end{array}$$ (14)

The values of *T* chosen were 1 h (the change since the last measurement), and also 2 h and 4 h, to reflect the same span of data used in the multi-hour convex hull area time series transformation.

### Inferring interactions

#### Cross-correlation

The cross-correlation between two time series for a given lag *τ* is the correlation between the first time series and the second offset by *τ*. This is given by:

$$\begin{array}{ccc} \rho_{XY}\left(\tau \right) & = & \frac{\sum_{t=1}^{n-\tau }(x_{t}-\bar{x})(y_{t+\tau }-\bar{y})}{\sqrt{\sum_{t=1}^{n-\tau }{\left(x_{t}-\bar{x}\right)}^{2}\sum_{t=1}^{n-\tau }{\left(y_{t}-\bar{y}\right)}^{2}}}, \end{array}$$ (15)

where *x*_*t*_ and *y*_*t*_, are the observed values of *X* and *Y* respectively and $\bar{x}$ and $\bar{y}$ their respective sample means.

We used cross-correlation as a measure to test for correlation between two of the scalar time series generated. Correlation is not causality, however, evidence of correlation is suggestive of possible interactions. To test for cross-correlation, the time series pairs have to be free of autocorrelation. This was undertaken by fitting an ARIMA model to each time series and taking the residuals. This was done in R software using the forecast package, which used the Akaike information criterion (AIC) for model selection [48–50]. The significance of cross-correlation values was tested non-parametrically by constructing a null distribution from the cross-correlations calculated when one of the time series had its order randomized. For example, when applied to the time series constructed for each of two species, it could be interpreted as interaction between the species causing one to modify the behavior of the other after *τ* observations of the behaviour of the other. If significant values exist only for one sign of *τ* (i.e. only negative or positive values of *τ* are significant) this could indicate a directional interaction, possibly causal, where one species is driving the behaviour of the other but not vice-versa.

#### Granger causality

According to definition 1 of [26] (p. 428), a time series “*Y*_*t*_ is causing *X*_*t*_ if we are better able to predict *X*_*t*_ using all available information than if the information apart from *Y*_*t*_ had been used”. We note that inherent in this assertion is that the behaviour of *X*_*t*_ can be predicted using past behaviour of *X*_*t*_. To be able to assess if *Y* Granger-causing *X* the Granger Causality Index (GCI) is defined as the log of the ratios between the variances in the error terms of a model using only *X* as a prediction of *X* (restricted model) compared to a model using *X* and *Y* for prediction of *X* (unrestricted model).

Defining the restricted model (no *Y*) as:

$$\begin{array}{ccc} x_{t} & = & \sum_{i=1}^{q}a_{i}x_{t-i}+e_{R,t}, \end{array}$$ (16)

where *q* is the order, or number of terms, of the model and *e*_*R*,*t*_ is the error term for the restricted model.

Then defining the unrestricted model as:

$$\begin{array}{ccc} x_{t} & = & \sum_{i=1}^{q}a_{i}x_{t-i}+\sum_{i=1}^{q}b_{i}y_{t-i}+e_{U,t}, \end{array}$$ (17)

with *q* as before and *e*_*U*,*t*_ the unrestricted model error term. The GCI is defined as:

$$\begin{array}{ccc} GCI_{Y\to X} & = & \ln\frac{\mathrm{Var}(\hat{e_{R,t}})}{\mathrm{Var}(\hat{e_{U,t}})}. \end{array}$$ (18)

Clearly, if the GCI is greater than zero, then *Y* contains information that can better predict *X*, as the variance of the unrestricted model is lower. A significance test can be undertaken using an F-distribution, with the F-statistic given by:

$$\begin{array}{ccc} F & = & \frac{(SSE_{R}-SSE_{U})/q}{SSE_{U}/\left(\left(n-q\right)-2q\right)}, \end{array}$$ (19)

where *n* is the length of each time series, *SSE* is the sum of squared errors of the subscripted regression (restricted, *R*, or unrestricted, *U*). The significance value is given by the relevant critical value of the F distribution with *q* and *n* − 3*q* degrees of freedom. These tests were carried out in R using the lmtest package [48, 51].

To be able to gauge causality between two time series in the presence of a third, or additional time series, Granger causality was extended to the conditional case by [31]. This is the case we must consider when we examine interactions at the individual animal level. As for Granger causality, *Y* conditionally Granger-causes *X*, conditioning on *Z*, if *X* can be better predicted using previous values of *X*, *Y* and *Z* as opposed to just previous values of *X* and *Z*. The restricted model becomes:

$$\begin{array}{ccc} x_{t} & = & \sum_{i=1}^{q}a_{i}x_{t-i}+\sum_{i=1}^{q}A_{i}z_{t-i}+e_{R,t}. \end{array}$$ (20)

While the unrestricted model is:

$$\begin{array}{ccc} x_{t} & = & \sum_{i=1}^{q}a_{i}x_{t-i}+\sum_{i=1}^{q}b_{i}y_{t-i}+\sum_{i=1}^{q}A_{i}z_{t-i}+e_{U,t}. \end{array}$$ (21)

The value of the conditional Granger causality index for *Y* causing *X* (*CGCI*_*Y*→*X*|*Z*_) is the same as in the two time series unconditional case. Its significance is also tested with an F-statistic [28], given by:

$$\begin{array}{ccc} F & = & \frac{(SSE_{R}-SSE_{U})/q}{SSE_{U}/\left(\left(n-q\right)-Kq\right)}, \end{array}$$ (22)

where *Kq* is the total number of coefficients in the unrestricted model. The significance is tested by the critical value of the F distribution with *q* and *n* − *q* − *Kq* degrees of freedom.

In the unconditional case, when applied to the time series at the species level, a significant Granger causality test for one species’ time series against that of the opposite species would indicate causality between the two. If the opposing test in the opposite direction were not significant, this would indicate directional causality, while if both directions were significant it would indicate a symmetric interaction where the behavior of one species influence the behaviour of the other and vice-versa. We note that we can only test for the presence or absence of inter-species interactions. Intra-species interactions are assumed as we Granger causality test against the restricted model.

In the conditional case, when applied to time series by individual animal, a significant conditional Granger causality test for one individual animal against another individual animal, conditional on all other animals present, would indicate causality between the two. This may indicate particular lead animals in the group, or be reflective of the broader underlying inter-species dynamics. Given the number of conditional Granger causality tests required for all of the animals present, using the results to expose underlying inter-species behaviour is made possible through the construction and analysis of complex networks.

### Complex network methods

A network is a set of vertices with connections between them called edges [52]. We followed a similar approach to that taken in [16] by constructing networks from the time series created using the methods described above. Each vertex represents an individual animal (sheep or deer), with connections being formed by a significant conditional Granger causality test p-value between the time series of that individual and the time series of another. (The time series of all other animals comprises the *z* terms in Eqs 20 and 21.) A network can be created for each transformation method of time series, and for each order *q* (1 ≤ *q* ≤ 10) of the conditional Granger causality test. To help minimize subsequent network analysis we collocated the information across data segments and each order *q*. In particular, we created summarized networks where edges were defined only if the conditional Granger causality was determined to be significant for at least three values of *q* and, for the real data sets, that this condition persisted across the experimental data segments. Thus in the 2006 data set the interaction had to be deemed significant in each of the three data segments and in the 2008 data set in each of the five segments (Table 1).

Two main network properties were used in the analysis of these summarized networks to extract information about species interaction. The first, degree, is the number of edges connected to a vertex, or the number of other vertices that vertex is directly connected to [52–54]. In directional networks, such as those constructed here, each vertex in turn has an in-degree (the number of incoming edges) and an out-degree (the number of outgoing edges) [52, 54]. The second, geodesic path length, is the length of the shortest path through the network from one vertex to another [52–54].

These two measures both offer an indication of how well connected an individual animal is to the group as a whole. For the understanding of the inter- and intra-species dynamics of the network we examined the differences between averages of out-degree and geodesic path length when examining sheep-sheep, deer-deer, sheep-deer edges.

## Results and discussion

### Testing data

#### Performance of time series by species methods

Tests for Granger causality were carried out for testing inter-species interactions and their direction (sheep → deer, deer → sheep), using each time series transformation method (CHA, MDNN, MDCM) and for test Data 1, 2 and 3 (Fig 1). A summary of the results is provided in Table 2. For the case where all animals were allowed to interact (Data 1), only MDCM and MDNN (*k* = 1, 6) were efficient to detect sheep → deer, and, deer → sheep interactions (*α* = 0.05). MDNN was also efficient to detect sheep → deer (*k* = 4) and deer → sheep (*k* = 5). MDCM was especially significant, with p-values of the order 10^−16^ in both directions for a Granger causality test of model order *q* = 3. This is unsurprising, since in the generated data it is the centre of mass of animals in the zone of attraction that individuals move towards. CHA performed poorly across the range of time intervals used to pool GPS fixes (1, 3 and 5 h), as it failed to detect any inter-species interaction. For test Data 2 (relationship matrix interactions: deer → deer, sheep → sheep, deer → sheep), there were significant Granger causality tests for deer → sheep for MDCM, MDNN (*k* = 6) and 1, 3, 5 h CHA. For sheep → deer there were only 5, non-expected by construction, significant tests out of 100, which we interpreted as type I errors. For the same species only interaction data (Data 3), most Granger causality tests were not significant for all of the time series creation methods. Indeed, for some model orders and some functions there were only 12 significant tests out of the 200 tests performed. This is again as expected since the relationship matrix for test Data 3 has no inter-species interactions built in. Although different transformations to scalar time series did not perform equally well to identify interactions, and in general, the case of no interactions were easier to detect than interactions, the methods were able to distinguish no interaction from interaction and determine the direction in the test data, suggesting it is valid to apply them to the real data.

#### Performance of time series by individual animal methods

For *k*^*th*^ nearest neighbour distance and distance from past position time series transformation methods, the summary networks created for all three test data sets were disconnected with few edges. Since the model used to create the data uses a constant speed for each animal, it can be expected that the distance from past position is not meaningful. The *k*^*th*^ nearest neighbour distance not producing many significant values is unexpected since the mean *k*^*th*^ nearest neighbour distance was useful in the time series by species case.

The networks created using the centre of mass distance and the Voronoi tessellation area transformation methods possessed large connected components, however, they were not able to replicate the network structures as defined by the relationship matrices (i.e., either all vertices connected (Data 1), all deer-deer, sheep-sheep and deer-sheep connections, but no sheep-deer edges (Data 2), and vertices only connected to the same species (Data 3)). This was mainly due to a much lower number of connections detected by the methods. We quantified this by calculating the average out-degree of the vertices in the reconstructed networks. For the centre of mass distance networks the average out-degree was 7.03, 6.52 and 6.78 for test Data 1, Data 2 and Data 3 respectively (for 40 animals, 20 deer + 20 sheep, Data1, 2 and 3 structure fully recovered would have average out-degree of 39, 29 and 19, respectively). The respective out-degrees for test Data 1, Data 2 and Data 3 for the Voronoi tessellation networks were 8.50, 7.88 and 7.46.

While the correct overall network structure could not be inferred from the transformed time series and Granger causality methods, broader results can be. In the all connected case (Data 1) the mean intra-species out-degree (the mean number of connections an animal has with members of the same species) for the three centre of mass distance networks (Eq 13) was 3.59, while the mean inter-species out-degree was 3.43 (a difference of 4.5%). This suggests detection of similar levels of inter- and intra-species interactions, which is the case. In the intra-species interaction only case (Data 3), the mean inter-species out-degree for the centre of mass distance networks was 34% lower than the mean intra-species out-degree. This captured the level of inter-species interaction compared to intra-species interaction, in this case, zero by design.

**Fig 3:**
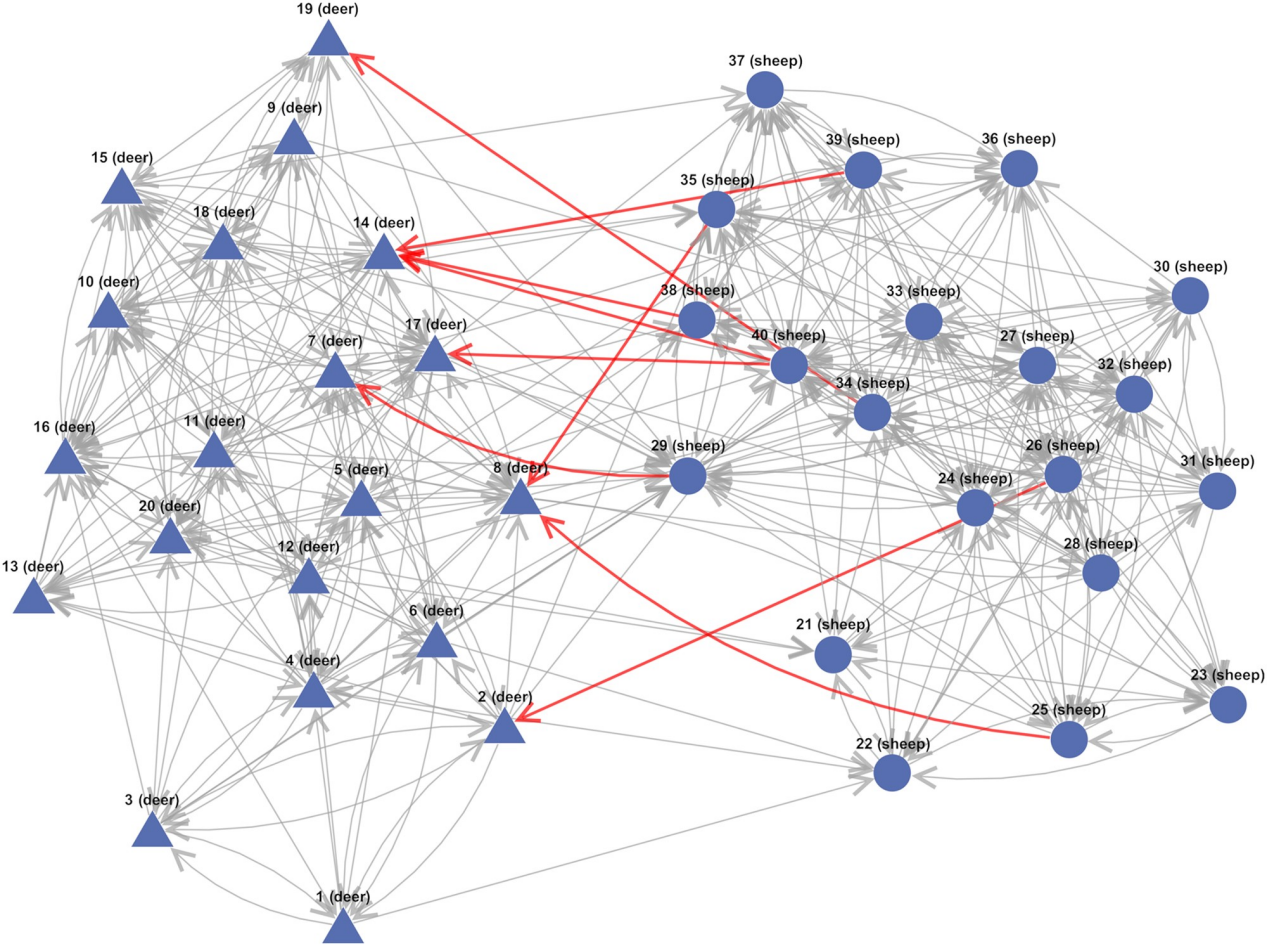
Simulated data example network. Inferred network for the Voronoi tesselation area transformation applied to test Data 2. Triangles denote vertices corresponding to deer and circles denote those vertices corresponding to sheep. Directed edges represent tail vertex Granger causing head vertex. In test Data 2 there should be no sheep → deer edges; only nine have been incorrectly inferred (red arrows). Although the exact relationship matrix for Data 2 has not been replicated, the overall propensity of inter and intra interactions have been captured, as reflected by the relative average out-degree of the different connection types (Table 3).

For the Voronoi tessellation area calculated using the same species networks, the mean number of deer a sheep was connected to in the intra-species interaction only for deer case (Data 2) was 0.45, while for deer-sheep connections the mean was 1.75. In the all connected case (Data 1) these numbers were comparable (differing by 11%). Thus the Voronoi tessellation area of the same species may be able to determine the direction of inter-species interaction, if it existed. In Table 3 we summarize the results of average out-degree of the summarized networks for the different types of connection. An example network structure which is informative of the capability of the approach for test Data 2 is shown in Fig 3.

### Actual data

#### Scalar time series by species

**Fig 4:**
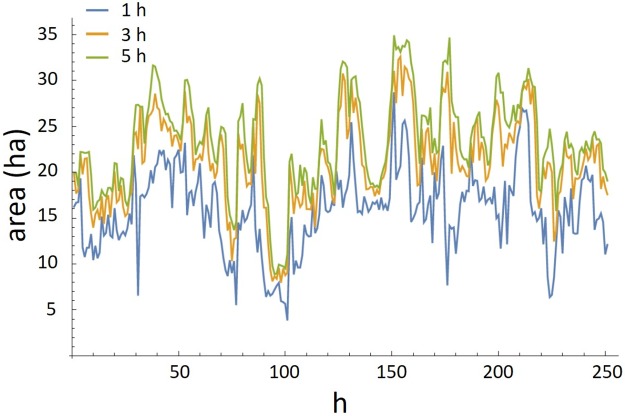
Time series of CHA. Convex hull area time series for sheep using time intervals of 1 h, 2 h and 3 h. Starting time 04:00 h on 20th of October 2006. Multi-hour intervals provides a greater area and are smoother than convex hulls calculated at 1 h interval.

**Fig 5:**
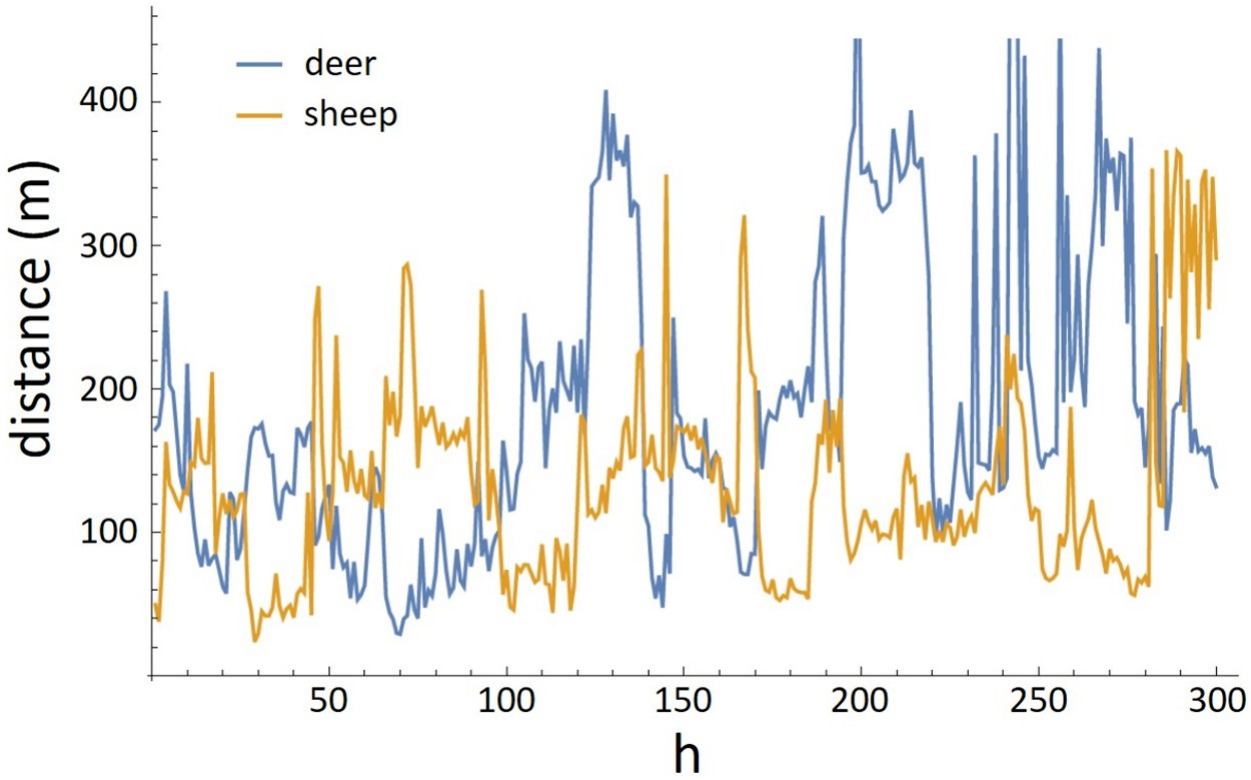
Time series of MDNN. Time series of mean 5^*th*^ nearest neighbour distance for sheep and deer. First 300 h, starting time 15:00 h on 5th of October 2006.

The transformations outlined in Table 2 were applied to the experimental data and species interaction was tested for using cross-correlation and Granger causality tests. Examples of CHA time series using time intervals of 1, 2 and 3 h, and MDNN (*k* = 5) are shown in Figs 4 and 5.

**Fig 6:**
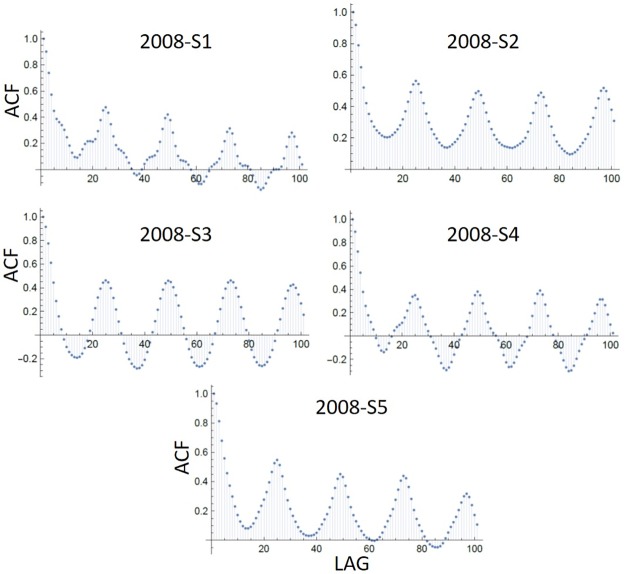
CHA autocorrelation. Autocorrelation function of the 5h CHA over 100 hours/lag for the five data segments of 2008 in Table 1, showing the evident daily 24 h cycle.

**Cross-correlation**—Prior to calculating cross-correlation the autocorrelation of the transformed time series was first calculated and then removed (i.e. pre-whitened). All transformed time series exhibited significant autocorrelation, showing a 24 h circadian cycle (Fig 6). However, in general, the cross-correlation of the pre-whitened time series showed no consistency of significant correlation structure.

**Fig 7:**
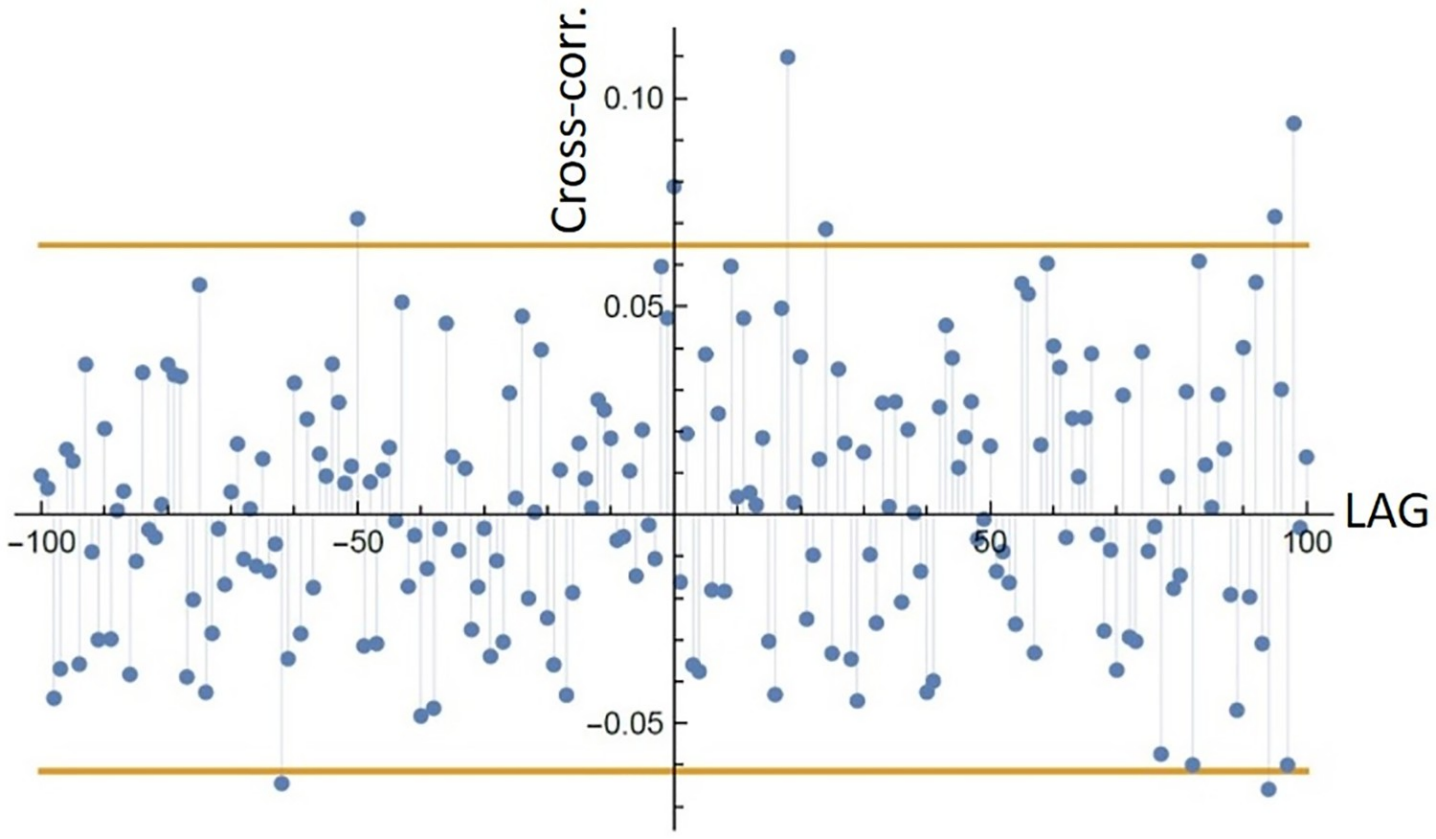
MDNN cross-correlation. Cross-correlation for the average distance to the *k* = 2 nearest neighbour transformation methods for lags between -100 and 100 h (data segment: 2006-S1). A significant cross-correlation for positive lag would indicate sheep influencing deer (sheep → deer), while for a negative lag it would indicate deer influencing sheep (deer → sheep). The bounds of the 95% confidence region are plotted horizontally in yellow, calculated non-parametrically by randomizing the order of the sheep time series and calculating the correlation 10000 times.

This indicates that there is no interaction between the two species or, more likely, cross-correlation between species time series subject to the transformation methods failed to detect any relationship interaction. This lack of structure is shown in Fig 7.

**Granger Causality**—Granger causality tests on pairs of transformed time series after applying a particular transformation method (Table 2) and with values of *q* = 1 to 10 were used to assess deer → sheep, sheep → deer. If both of these types of interactions were significant then this implied deer ↔ sheep causality. We present the results for the CHA transformation methods for the eight data segments of Table 1. In Table 4 we show for the CHA transformation methods for the eight data segments of Table 1 the calculated p-values for each order *q* = 1 to 10 in the Granger causality tests. The 3 h and 5 h convex hull area time series revealed significant deer ↔ sheep interactions for greater than three values of *q* for four and five of the eight time series segments, respectively, but no deer ↔ sheep interaction for 1 h interval (Table 4). Asymmetric interactions deer → sheep for greater than three values of *q* were detected for three time series segments at 1 h interval, and sheep → deer interactions for one segment at 1, 3 and 5 h intervals (Table 4). These results are summarized in Table 5 which also includes summarized interactions obtained using the other time series transformation methods. At a species group level the data supports the hypothesis of symmetrical inter-species interaction, where sheep influences deer behaviour and vice-versa.

**Table 4:** Granger causality test p-values (for order of model from *q* = 1 to *q* = 10) for 1h, 3 h and 5 h convex hull area. Significant values are bold highlighted. See Table 2 for arrow acronyms.

Interval	Interaction	Segment	*q*									
			1	2	3	4	5	6	7	8	9	10
1	*D* → *S*	2006-S1	0.261	**0.035**	0.054	0.113	0.175	0.233	0.304	0.262	0.371	0.478
		2006-S2	0.383	0.876	0.320	0.518	0.259	0.339	0.257	0.353	0.437	0.512
		2006-S3	0.207	0.121	0.210	0.235	0.291	0.392	0.530	0.564	0.514	0.655
		2008-S1	0.407	0.375	0.471	0.600	0.195	0.218	0.182	0.237	0.166	0.197
		2008-S2	**0.018**	**0.044**	0.080	0.067	**0.007**	**0.000**	**0.000**	**0.000**	**0.000**	**0.001**
		2008-S3	**0.035**	**0.003**	**0.006**	**0.001**	**0.002**	**0.002**	**0.001**	**0.002**	**0.004**	**0.005**
		2008-S4	0.209	0.448	0.208	**0.005**	**0.013**	**0.024**	**0.049**	0.066	0.098	0.106
		2008-S5	**0.006**	**0.023**	0.056	0.079	0.092	0.075	0.097	0.140	0.199	0.213
1	*S* → *D*	2006-S1	**0.002**	**0.012**	**0.045**	0.064	0.087	0.132	0.143	0.201	0.277	0.229
		2006-S2	0.147	0.372	0.581	0.742	0.834	0.287	0.388	0.330	0.326	0.353
		2006-S3	0.728	0.859	0.744	0.728	0.406	0.523	0.330	0.399	0.537	0.551
		2008-S1	0.534	0.210	0.265	0.405	0.503	0.512	0.621	0.688	0.771	0.817
		2008-S2	0.062	0.243	0.364	0.145	0.051	0.098	0.083	0.093	0.131	0.161
		2008-S3	0.964	0.913	0.870	0.810	0.765	0.808	0.870	0.927	0.857	0.542
		2008-S4	0.882	0.895	0.911	0.260	0.131	0.237	0.309	0.248	0.244	0.347
		2008-S5	**0.049**	0.565	0.821	0.885	0.719	0.767	0.468	0.457	0.563	0.652
3	*D* → *S*	2006-S1	0.789	0.847	0.682	0.383	0.774	0.883	0.846	0.345	0.225	0.299
		2006-S2	0.845	0.967	0.353	0.185	0.270	0.383	0.531	0.304	0.349	0.301
		2006-S3	0.153	0.215	0.507	0.530	0.718	0.812	0.605	0.599	0.661	0.751
		2008-S1	0.892	**0.008**	**0.008**	**0.000**	**0.000**	**0.000**	**0.000**	**0.000**	**0.000**	**0.000**
		2008-S2	0.079	**0.036**	**0.004**	**0.000**	**0.000**	**0.000**	**0.000**	**0.000**	**0.000**	**0.000**
		2008-S3	0.214	0.054	0.147	**0.000**	**0.000**	**0.000**	**0.001**	**0.001**	**0.001**	**0.002**
		2008-S4	0.583	0.618	0.663	0.463	0.648	0.754	0.841	0.877	0.787	0.860
		2008-S5	**0.009**	**0.003**	**0.005**	**0.000**	**0.000**	**0.000**	**0.000**	**0.000**	**0.000**	**0.000**
3	*S* → *D*	2006-S1	**0.041**	**0.007**	**0.004**	**0.004**	**0.003**	**0.007**	**0.011**	**0.018**	**0.030**	**0.039**
		2006-S2	0.264	0.237	0.352	0.407	0.406	**0.025**	**0.028**	**0.027**	0.053	0.056
		2006-S3	0.570	**0.020**	**0.042**	0.068	0.070	0.100	0.073	0.103	0.159	0.206
		2008-S1	0.634	**0.000**	**0.000**	**0.000**	**0.000**	**0.000**	**0.000**	**0.000**	**0.000**	**0.001**
		2008-S2	**0.007**	**0.000**	**0.000**	**0.001**	**0.005**	**0.008**	**0.014**	**0.017**	**0.027**	**0.041**
		2008-S3	0.507	**0.010**	**0.013**	**0.024**	0.090	0.072	0.059	0.076	0.086	0.027
		2008-S4	0.801	**0.002**	**0.001**	**0.003**	**0.007**	**0.013**	**0.021**	**0.028**	**0.044**	0.060
		2008-S5	0.051	**0.000**	**0.000**	**0.000**	**0.000**	**0.000**	**0.000**	**0.000**	**0.000**	**0.000**
5	*D* → *S*	2006-S1	0.705	0.398	0.547	0.350	0.683	0.803	0.865	0.328	0.269	0.406
		2006-S2	0.794	0.917	0.054	0.105	**0.045**	**0.041**	0.051	0.105	0.137	0.061
		2006-S3	0.235	0.205	0.273	0.353	0.413	0.419	**0.040**	**0.041**	**0.043**	0.059
		2008-S1	0.436	0.095	**0.000**	**0.000**	**0.000**	**0.000**	**0.000**	**0.000**	**0.000**	**0.000**
		2008-S2	0.123	**0.017**	**0.028**	**0.000**	**0.000**	**0.000**	**0.000**	**0.000**	**0.000**	**0.000**
		2008-S3	0.267	0.058	0.173	**0.008**	**0.001**	**0.001**	**0.001**	**0.001**	**0.001**	**0.001**
		2008-S4	0.858	0.707	0.697	0.507	0.529	0.664	0.545	0.622	0.546	0.642
		2008-S5	**0.003**	**0.001**	**0.001**	**0.000**	**0.001**	**0.000**	**0.000**	**0.000**	**0.000**	**0.000**
5	*S* → *D*	2006-S1	0.229	**0.008**	**0.012**	**0.001**	**0.003**	**0.006**	**0.011**	**0.017**	**0.012**	**0.013**
		2006-S2	0.461	0.605	0.553	0.687	0.513	0.068	0.094	0.126	**0.041**	0.057
		2006-S3	0.707	**0.001**	**0.002**	**0.005**	**0.008**	**0.007**	**0.009**	**0.011**	**0.017**	**0.026**
		2008-S1	0.776	**0.000**	**0.000**	**0.000**	**0.000**	**0.000**	**0.000**	**0.000**	**0.000**	**0.001**
		2008-S2	**0.020**	**0.000**	**0.000**	**0.000**	**0.000**	**0.001**	**0.001**	**0.003**	**0.003**	**0.004**
		2008-S3	0.442	**0.008**	**0.010**	**0.015**	0.066	0.190	0.245	0.351	0.446	0.074
		2008-S4	0.221	**0.009**	**0.023**	0.051	0.116	0.132	0.178	0.221	0.283	0.344
		2008-S5	0.681	**0.001**	**0.002**	**0.000**	**0.000**	**0.000**	**0.000**	**0.000**	**0.000**	**0.000**

**Table 5:** Detected species interactions across data segments for the various time series transformations methods using Granger causality. Interaction was judged to be detected if the p-values calculated were significant for at least three model orders (for *q* = 1 to 10) of the Granger causality tests. *A* → *B* means *A* Granger causes *B*, *A* ↔ *B* means symmetric interaction,—means less than three model order were significant hence no interaction detected.

	Timespan	*k*	2006-S1	2006-S2	2006-S3	2008-S1	2008-S2	2008-S3	2008-S4	2008-S5
CHA	1		*S* → *D*	—	—	—	*D* → *S*	*D* → *S*	*D* → *S*	—
	3		*S* → *D*	*S* → *D*	—	*S* ↔ *D*	*S* ↔ *D*	*S* ↔ *D*	*S* → *D*	*S* ↔ *D*
	5		*S* → *D*	—	*S* ↔ *D*	*S* ↔ *D*	*S* ↔ *D*	*S* ↔ *D*	—	*S* ↔ *D*
MDNN		1	—	—	—	—	*D* → *S*	—	—	—
		2	—	—	—	—	*D* → *S*	*D* → *S*	*D* → *S*	—
		3	—	—	—	—	—	—	—	—
		4	—	—	—	—	*S* → *D*	—	*D* → *S*	*S* → *D*
MDCM			—	—	*D* → *S*	—	*D* → *S*	*D* → *S*	—	*S* → *D*

The p-values for the other time series transformation methods (MDCM, MDNN for 1 ≤ *k* ≤ 4) showed that where significant Granger causality tests did occur, they mostly revealed deer → sheep interaction (*n* = 8) against sheep → deer (*n* = 4) in five time segments but no symmetric deer ↔ sheep interaction (Table 5).

#### Scalar time series by individual animal

**Fig 8:**
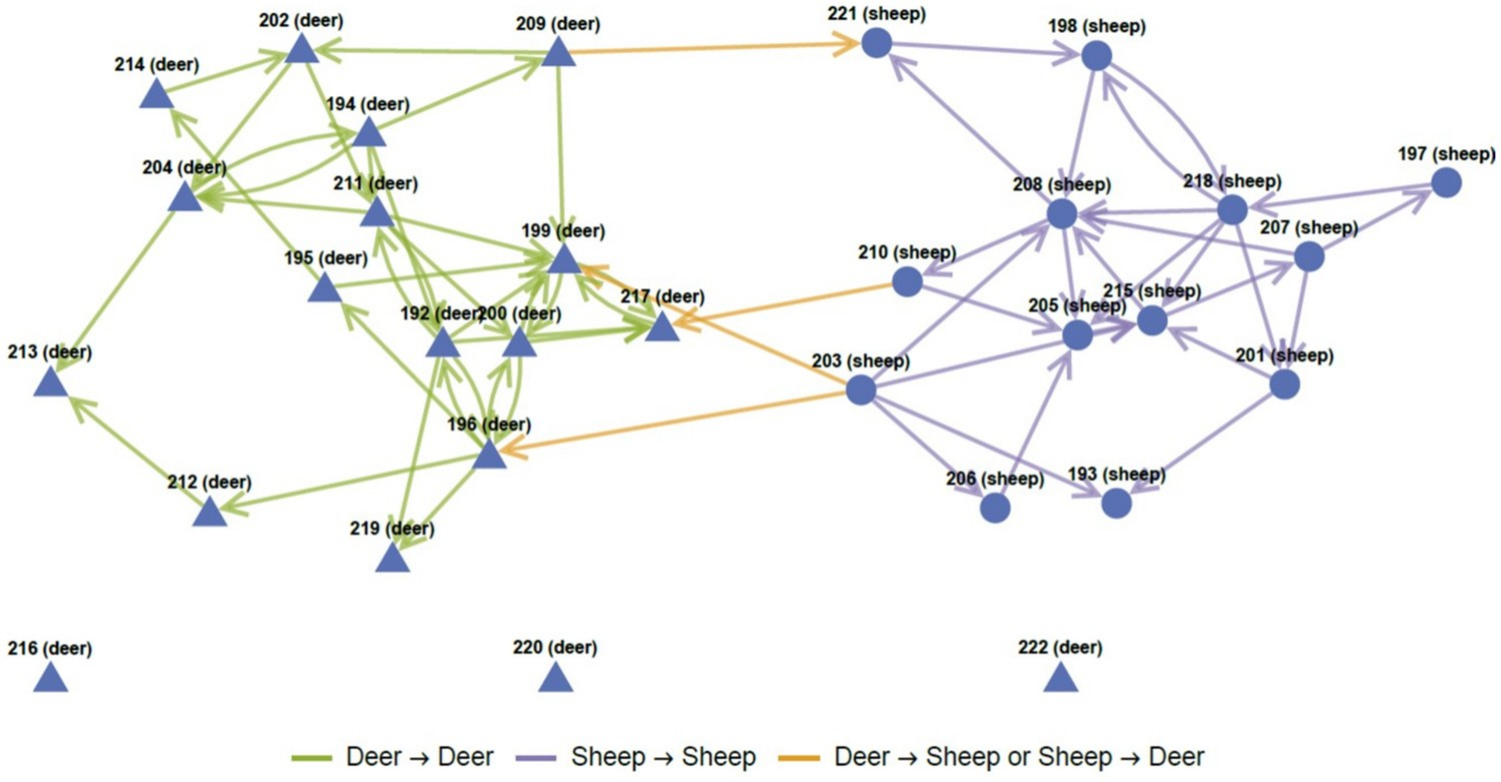
MDNN network. A visualization of the summarized network produced using conditional Granger causality to create edges using the distance to the *k* = 4 nearest neighbour of the same species time series for each individual animal within the 2006 (S1-S3) data set. Deer are represented by triangles, and sheep are represented as circles.

For both the 2006 and 2008 data sets most of the time series transformation methods performed well in the sense that they created networks with a reasonable number of edges. That is, the Granger causality tests between transformed time series of pairs of individual animals revealed significant p-values for at least three model orders *q*. Furthermore, such significance persisted across the data segments (segments 1 to 3 for 2006 data and segments 1 to 5 for 2008 data sets). Thus an edge between vertices representing such pairs of animals was created and consequently these summary networks possessed a large connected component. An example of such a summary network is shown in Fig 8, where the time series transformation method was the *k* = 4 nearest neigbour in the case of the three segments of 2006 data. We observe from this summary network that most of the detected interactions are intra-species as there are far more connections of the sheep-sheep and deer-deer type and only four inter-species connections and three of these are sheep → deer. We note that there are three deer with no connections and their behaviour with respect to their *k* = 4 nearest neighbour is completely independent.

The summary networks for the VTA transformed time series in both 2006 and 2008 data sets also had a high number of connections. Similarly, summary complex networks from time series created by computing DCM using Eq 13 for either the same species, the opposite species or of both species produced highly connected networks. The time series from the DNN (particularly for 3 ≤ *k* ≤ 6) also had many significant conditional Granger causality p-values, of which Fig 8 is a *k* = 4 example. To the contrary, the DPP time series transformation method produced disconnected networks without large connected components, with many vertices having a degree of zero (no incoming or outgoing edges, cf. the three isolated deer in Fig 8).

**Fig 9:**
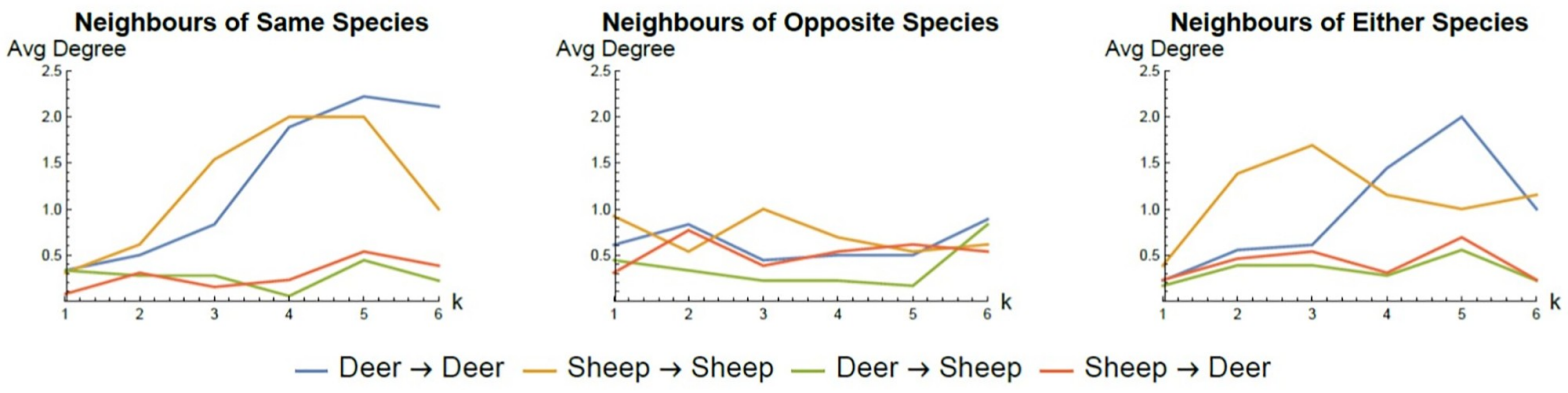
MDNN network degree. The average degree for different types of inferred interaction in summarized networks of the 2006 (S1-S3) data set created using the *k* nearest neighbour transformation methods and conditional granger causality tests. The typically higher value for intra-species interaction suggests this is the stronger driver for animal behaviour.

**Fig 10:**
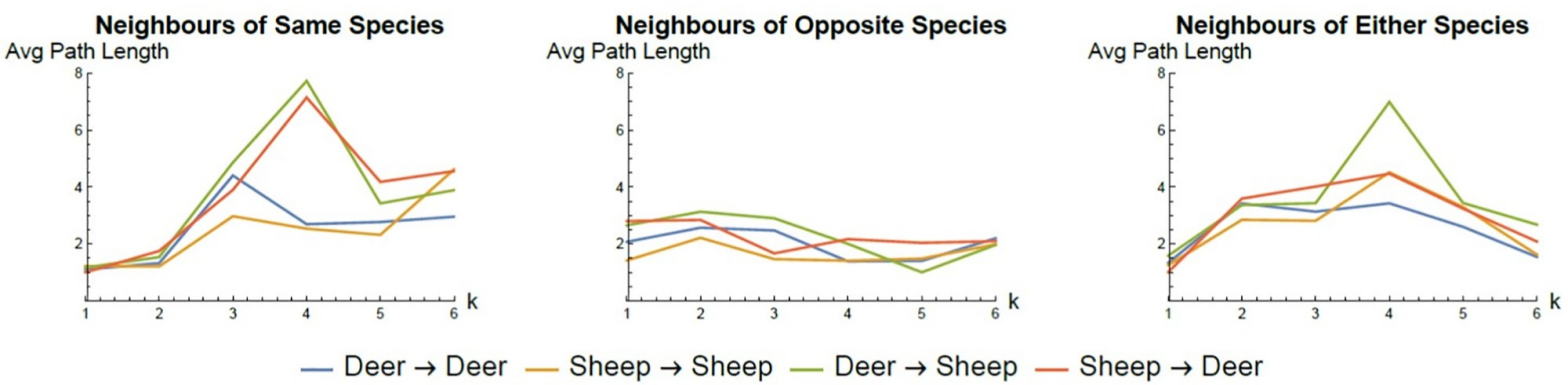
MDNN network shortest path length. The average geodesic (shortest) path length for different types of inferred interaction in summarized networks of the 2006 (S1-S3) data set created using the *k* nearest neighbour transformation methods and conditional granger causality tests. Analogous to degree the typically lower value for intra-species interaction suggests this is the stronger driver for animal behaviour.

We analyzed the summarized networks for each time series transformation method by examining the average degree and the average geodesic (shortest) path length between vertices in the networks (see Figs 9 and 10 respectively for the *k* nearest neighbour transformation method using the same, opposite or combined species in the 2006 data). The summarized networks for the 2008 data set exhibited similar profiles. Several general trends are evident suggestive of the possible interaction behaviour between sheep and deer in these experiments. We observe that the average degree for intra-species edges (deer-deer, sheep-sheep) is higher than for inter-species (deer-sheep, sheep-deer) edges. This suggests that inter-species interaction is less dominant than intra-species interaction. Similar conclusions can be drawn from the geodesic path lengths. Typically, the shortest paths between intra-species individuals are lower than inter-species shortest paths suggesting more densely connected intra-species subgraphs compared to inter-species connections. Consequently, the interaction between an individual and members of its own species is stronger than the interaction between individuals and members of the opposite species.

## Discussion

### Performance of methods on testing data

Our results clearly show that the methods assessed differ in their efficiency to detect animal interactions and their symmetry. The main factor that characterizes their efficiency is whether interactions take place between groups or individuals, and all methods are more efficient to detect interaction between species than between individuals. In our data this is unsurprising as the complexity of individual interactions was higher than the inter-species interactions (40 individual animals versus 2 species). Although fair comparisons between methods are not straightforward due to their different nature, CHA, MDNN and MDCM methods applied to inter-species relationship matrices performed well.

MDCM was able to detect all different interactions and symmetry embedded in the simulated animal movement, followed in efficiency by MDNN and CHA methods. MDNN performance depended on the number of nearest neighbours included in the transformation method, and although the response was not linear, when the number of neighbours was *k* = 6 MDNN was able to correctly detect all interactions and their symmetry (Table 2). CHA performed well for different timespans (1, 3, 5 hours) on matrices that contained interactions driven by one species, and in the case where there were no interactions between species, but CHA failed to detect symmetric interactions (i.e. when each species were allowed to modify the movement behaviour of the other). As a result of this issue CHA was the worst performer of the three methods.

One of the issues with convex hull areas is its high dependence on specific individual animals that segregate far away from the main core of the group. This high dependence on outliers means that the convex hull area may not be reflective of the state of all members of the group. In real experiments, missing data for one or two influencing animals due to temporary GPS failure may also have a large effect on the convex hull area. The poor performance of CHA in the test data may also be due to the simplifications of the model (see below: Assessing interactions on experimental data). [21] noted that the Couzin model could not replicate the cluster-disperse cycle in animal movement that drove changing convex hull areas in their experiments. MDCM performed best probably, in part, due to the inherent smoothing intra-species effect of the transformation because distances are taken from the species center of mass, which minimizes the effect of spatial outliers.

The four methods applied to infer interactions at an individual level (DNN, DPP, DCM, VTA) performed poorly in uncovering the exact structure of the test data interaction matrices. The transformations DPP and DNN, in particular, were unexpectedly poor. DPP is based on the distance an animal moved from a previous position, and in view of our results it seems a poor criterion to capture the movement of the simulated data movement, given that the networks created were highly disconnected. [20], however, used this criterion to reconstruct a state-space to model the flocking behavior in flight of pigeons with nonlinear basis functions to capture interaction behaviour. The fact that DNN and DPP are bad performers may be an indication that linear models within the Granger causality tests are not adequate to capture the true behaviour at the resolution of individual animals. This shortcoming of Granger causality has been recognized and palliative measures have been suggested to better deal with nonlinearity. These include information theory measures such as transfer entropy [55, 56] and state-space methods [30, 57]. The other two methods applied (DCM, VTA) enabled the creation of networks with large connected components but not as expected by the construction rules of the simulated relationship matrices (see, Fig 3 for VTA on test Data 2). However, they were able to capture the levels of intra- and inter-species interactions embedded in the relationship matrices, which again reinforce the idea that the methods used perform better to infer inter-species than inter-individual relationships.

### Movement model limitations

Before discussing the results of applying the different methods to our experimental data on sheep and deer movement, we should highlight the limitations of the simulation model used here to replicate actual sheep and deer movements. We reiterate that the purpose of the model was to investigate if the structure of the embedded relationship matrices could be recovered using our methods and was not to propose a realistic animal movement model. Nevertheless, it is instructive to discuss some of the shortfalls of the model and its assumptions so that better and more appropriate models can be proposed in future.

The model makes many simplifications in its structure, such as not allowing for different animal states, e.g. grazing activity [19], changes in distance travelled, temporal simultaneous orientation and attraction, or orientation and repulsion [7, 21]. In reality there may be different values of movement speed (*v*) based on species, underpinned by differences in body size and energy requirements [58, 59], and different values of *r*_*r*_, *r*_*o*_ and *r*_*a*_ for each species [60], and for inter- and intra-species interactions [9]. Additionally, updating of the position based only on the direction, velocity and previous position does not allow for accounting for the effect of physical environmental factors on behaviour, such as the circadian rhythm. However, the model used is general enough to capture basic animal movement behaviours in order to test the effectiveness of the approach taken to produce scalar time series by either species or individual animals from the data.

One of the issues with recording time series of animal movement is the selection of the appropriate time interval at which animal positions are collected [17, 61]. To infer different behaviours one might need the use of different time intervals, or the same behavior might need different time intervals with respect to different species. When it is feasible to select different time intervals of data gathering, a way to avoid making assumptions on the most appropriate interval is to assess the robustness of the results against interval changes. [17] assessed how GPS fixes acquisition interval was instrumental to capture complex social dynamics in sheep movement around parturition. At short time intervals their models were able to predict how likely a ewe changed its dynamic social unit, but the longer the interval the less reliable was the prediction. Thus an additional task to ask of any simulated model is to test the effect of different sampling schemes on the proposed methods.

[21] used convex hull area as a measure for the clustering and dispersion behaviours of sheep. They found that sheep slowly disperse (increasing convex hull area) before rapidly forming into a tight cluster (sudden decrease in convex hull area) before slowly dispersing again with a period of around 10 minutes. Our experimental data in use is hourly and we sampled the simulation to mimic this situation, and so social structures with shorter time-span will not be adequately captured in the CHA time series transformation measure. This effect also means that any time-span trends that occur in the spread of sheep due to the behaviour of the other species will have a large amount of noise depending on where in the cluster-disperse cycle of sheep the data was captured.

Another issue to consider is the effect of spatial scale on movement behaviour. Under spatial restrictions, such as experimental plots or in larger areas where animals are attracted by preferred resources [12, 62], frequent interaction between species that compete for resources is likely and the need for finer resolution (more frequently sampled) time series is less crucial than in conditions when species interact with less frequency. The flexibility of GPS technology allows for very short intervals between fixes but at a cost of battery life, so careful planning is needed to balance detailed time series and battery life, especially when GPS time deployment should be maximized because animal handling (to recharge collars) is logistically difficult or impossible. These considerations are important not only for logistical experimental design but in meta-analysis that use results from studies carried out in very different conditions, possibly making comparison difficult or even incomparable.

### Assessing interactions on experimental data

The results clearly indicate that interaction between sheep and deer is more complex than expected under the traditional framework of niche partitioning in the guild of large herbivores [63, 64]. Expected predictions of the interaction between two grazing species are based on the functional response: the relationship between the two competing terms that define intake rate. These are bite mass and bite rate [65]. [66] created a model in which coexistence between grazers differing in body size was possible if a single-resource type offered variation in height structure. Large grazers could facilitate food availability for smaller species as intake rate varies between species that differ in bite width and bite depth [67]. On these premises it would be predicted that in our data set larger red deer (92 kg) graze on a higher sward than smaller sheep (47 kg) [9] facilitating grazing for sheep, and consequently, deer driving sheep movement. However, with reference to Table 5 which itself is a summary of Table 4 for CHA and other time series tables (not shown), at the species-level analysis this prediction was only supported by 17% of the tests (11 of the 64 entries). Almost 13% of these tests across the different data segments and time series transformation methods supported the opposite, i.e., sheep drives the movement of deer. Moreover, 14% of the tests show that the interaction between sheep and deer is symmetric, and in 56% of the cases no significant inter-species interaction could be detected. It is worth noting that the methods showed a bias for non-detection on the test data and the figure of 56% is reported assuming all methods were equally good at detecting interaction if it exists. We have also chosen to report the results of all methods investigated and not just the “good” or “bad” methods which would lower or increase the success rate.

It is relevant to note the fact that within each transformation method the nature of the inter-species interaction was not consistent across different time series segments, nor did we observe consistency of the methods performance between the testing and the actual data. This suggests three main explanations, (i) the movement drivers of the actual data are more complex than those used to create the testing data, (ii) the driving dynamics of inter-species interactions change across time, a possible seasonal and resource effect, and (iii) the levels of interaction forces vary across individuals of the same and different species, as corroborated by the lack of consistency in the nature of the interactions across MDNN k-values, and by the by-individual methods. In general, the inconsistency of the methods on the real data and as supported by the testing data results suggests the weakness of the approach is the number of false negatives—not always detecting a genuine interactions—as opposed to the number of false positives—detecting a spurious interaction.

Since more than half of the tests carried out did not detect any significant interaction it may be interpreted as sheep and deer do not interact strongly. Although this is possible, this interpretation should be taken with precaution. Consider the following situation in which there is behavioural synchronization between species, for example, in rumination bouts or night resting. The methods would be more likely to detect bouts of no significant interaction since little new information to predict one species behaviour is available from the behaviour of the other species. On the other hand if the within species behaviour is highly synchronized, this can also produce bouts of no significant interaction that depend only on the behaviour of one species, for example, when one species lie down regardless of the other moving away.

Sheep and red deer commonly graze in large numbers within grass-heather dominated mosaic areas in northern Britain and other parts of Europe [68, 69]. In a grass-heather mosaic and under controlled conditions [70] found that both red deer and sheep consumed similar proportions of grass and heather year round, even when grass availability declined. Only small differences in the use of the mosaic were found with sheep showing preferences in the use of small grass patches whereas deer make spatial use of grass and heather patches more evenly. Although [70] did not carry out a spatial analysis to assess for species interaction they suggested that due to their tight range overlap the overall patterns of foraging behaviour by sheep and deer were little affected by the presence or absence of the other species. Our results support these findings, as we have not found clear evidence or consistency across different methods of analysis that one species drives the movement behaviour of the other species.

Our actual data is affected by two main factors of noise, (i) the number of GPS marked animals varied between some of the time series segments (Table 1), (ii) not all animals in the experimental plot were GPS marked. The latter was especially true in the case of sheep when on average there were 31% of individuals marked vs. 80% in deer. This was a compromise in the experimental design between a large size plot, similar grazing densities to the ones found in open hill farming systems and the number of GPS units available. The effect of the variation in the number of animals across the experiment was controlled by creating different time series segments that were homogeneous in the number of animals and applying the methods to each segment separately. The fact that only part of the population was marked could not be controlled in the analyses, but we assumed that in most real situations only a small part of the population can be marked and conclusions have to be derived from the data.

Our results suggest that methods which assess the interaction based on proximity, rather than on topological metrics, provide results easy to interpret in terms of behavioural interaction and can facilitate their incorporation on to layers of geographical information, such as vegetation communities, mosaic fragmentation, land cover, which adds extra information for the understanding of the factors that drive movement behaviour [9].

### Methods for aims

The lack of consistency of the results obtained across different methods, in both the testing and actual data, suggests that depending on the aims of the analysis different methods are better suited to uncovering different types of interactions than others. All transformations, as evidenced by the autocorrelation revealing a strong circadian rhythm, contain useful information and extract different features and aspects of the animal’s behaviour which may be useful in determining interactions. Furthermore, because of the different analytical requirements for the application of the methods, specific data properties will also determine the choice of methods to use. Based on our experience with the testing data and the experimental data we suggest if the aim is to unravel interactions between individuals or to uncover association between sub-groups of individuals within a population or within an assemblage of species, then individual-individual and individual-group distance based methods (DNN, DPP, VTA, DCM, Table 6) are more appropriate. Within these methods individual-individual based distance calculations require high quality datasets as the construction of their metrics are more sensitive to the presence of missing data and timescale acquisition time (DNN, DPP, Table 6). Datasets with missing data, noisy (outliers) or poor positional time schedule can be analyzed using methods that smooth these issues (VTA, DCM) because their metrics are based on the properties of the spatial distribution of the group unit, which can also be pooled across time if required. When assessing interactions between pre-established groups (social units, sexes, taxa, experimentally defined units) MDNN, CHA and MDCM are desirable methods (Table 6). MDNN is sensitive to data quality, especially to the presence of missing data and outliers, as it has an important individual distance component in their construction (i.e. distance to *k*-nearest neighbour), while CHA and MDCM can best mitigate this issue. However, the application of a number of methods is always recommended as this provides a more realistic picture of the complexity of the problem, whether consistent conclusions, or not, can be obtained across the different methods.

**Table 6:** Suggested use of time series transformation methods in relation to the type of interactions aimed and the quality of the data available.

	Aims	
Data quality	Individual interactions, sub-groups associations	Group, social, taxon interactions
Accurate, complete, appropriate timescale	DNN DPP	MDNN
Noisy, missing, unsuitable timescale	VTA DCM	CHA MDCM
